# Supplementary material for: Thermal sensitivity of soil microbial carbon use efficiency across forest biomes
Source: Nat Commun. 2024 Jul 25;15:6269. doi: 10.1038/s41467-024-50593-6 (PMC11272934; doi:10.1038/s41467-024-50593-6)
Supplement: Supplementary file 1 — Supplementary Information [file 41467_2024_50593_MOESM1_ESM.pdf]

Supplementary information for

Thermal sensitivity of soil microbial carbon use efficiency across forest biomes

Chengjie Ren, Zhenghu Zhou, Manuel Delgado-Baquerizo, Felipe Bastida, Fazhu Zhao, Yuanhe Yang, Shuohong Zhang, Jieying Wang, Chao Zhang, Xinhui Han, Jun Wang, Gaihe Yang, Gehong Wei

This file includes:

Supplementary Texts 1-2

Supplementary Tables 1 to 8

Supplementary Figures 1-7

Supplementary Text 1: Metagenomics analysis.

Soil DNA was extracted in triplicate from 0.5 g of fresh soil sample using the FastDNA spin kit for soil (MP Biomedicals, Cleveland, United States), following the manufacturer's instructions. The metagenome libraries were sequenced on an Illumina HiSeq 2000 to generate 150 bp paired-end reads at greater sequencing depth. The reads aligned to the human genome were removed, and the lengths were trimmed with Sickle. All DNA sequencing can be found on the National Center for Biotechnology Information (NCBI) website. To improve the reliability and quality of subsequent analysis, the sequencing reads were filtered as previously described (Ren et al., 2022), which included removing the adapter sequences, trimming the reads, and discarding the quality-trimmer reads that were below than 50 bp or containing N (ambiguous bases). For the utilization of high-quality reads, the megahit software (Li et al., 2015) was used to assemble the mixed sequence of all samples, and thus the larger database of contigs and Scaffolds were generated. It is noted that not all sample data are mixed and sequenced, instead, each sample is individually assembled first, then mixing the unused reads from the single spell together to obtain more contigs data. The clean data and assembly status were shown in Table S3, which have been showed in our recent study (Ren et al., 2022). The MetaGeneMark was used to predict the genes in the contigs (longer than 200bp), and the per-base coverage depth across all contigs was calculated by mapping raw reads from each sample (Zhu et al., 2010).

Supplementary Text 2: Quantitative PCR.

Microbial (bacterial and fungal) abundances were determined by realtime PCR of the 16 S rRNA and fungal ITS-1 genes, as previously described (Ren, et al., 2017). Specially, PCR amplification of the bacterial 16S rRNA and fungal ITS-1 region was conducted by using bacterial-and fungal-specific primer pairs. The 20 μl quantitative PCR (qPCR) reactions of bacteria contained 10 μl of EvaGreen 2X qPCR Master Mix, 0.5 μM of each primer, an environmental and standard DNA template and sterile ddH2O. While the 20 μl qPCR reactions of fungi contained 10 μl EvaGreen 2 X qPCR MasterMix, 0.3 μM of each primer, and environmental or standard DNA templates. Then, quantitative PCR for microbial (bacteria and fungi) were finally performed using a Bio-Rad C1000/CFX96 Thermocycler. All the qPCR reactions were run in triplicate with each DNA template and amplification efficiency of qPCR was 90% to 97% (R^2 > 0.99). Finally, the Bio-Rad CFX Manager Software, installed on Bio-Rad C1000/ CFX96 Thermocycler, was used to test the bacterial 16S rRNA and fungal ITS^{-1} copy numbers, the units of bacterial and fungal abundance is $\log_{10}$ copy number·g^{-1}. The ratio of fungi to bacteria (F: B) represented the ratio of fungal abundance to bacterial abundance.

References

Ren, C. J. et al. Microbial traits determine soil C emission in response to fresh carbon inputs in forests across biomes. Global Change Biology. 28, 1516-1528(2022).

Ren, C. J. et al. Response of microbial diversity to C: N: P stoichiometry in fine root and microbial biomass following afforestation. Biology and Fertility of Soils. 53: 457–468 (2017)

Li, D. H. et al. MEGAHIT: An ultra-fast single-node solution for large and complex metage-nomics assembly via succinct de Bruijn graph. Bioinformatics. 31, 1674–1676. (2015)

Zhu, W. H.et al. Ab initio gene identification in metagenomic sequences. Nucleic Acids Research. 38 (2010)

Supplementary Table 1 | Background information across forest biomes.

Site	Elevation (m)	Latitude (°)	Longtitude (°)	MAP (mm)	MAT (℃)	Climate types	Vegetation type
Mt. Maoer	600	45.41	127.71	629	3.10	Temperate continental	Coniferous and broad-leaved mixed forest
Mt. Dongling	1300	39.97	115.43	650	5.00	Warm temperate semi-humid	Deciduous broad-leaved forest
Fuxian	1035	36.11	109.63	580	7.40	Warm temperate	Deciduous broad-leaved forest
Huoditang	1526	33.43	108.45	1023	9.00	North subtropical humid	Deciduous broad-leaved forest
Maoxian	1625	31.79	104.71	486	11.00	North subtropical humid	Deciduous broad-leaved forest
Mt. Gongga	1726	29.65	102.11	1000	13.00	Subtropical	Evergreen deciduous broadleaf mixed forest
Mt. Ailao	2300	23.87	103.51	1086	18.30	Subtropical humid	Evergreen broad-leaved forest
Xishuangbanna	560	21.83	101.20	1500	21.60	Tropical humid	Rainforest
Jianfengling	820	18.71	108.91	2266	23.15	Tropical humid	Rainforest

Mt., mountain; MAT, mean annual temperature; MAP, mean annual precipitation. Source data are provided as a Source Data file

Supplementary Table 2 | Soil basic physio-chemical and biological properties across forest biomes.

	**Mt. Maoer**	**Mt. Dongling**	**Fuxian**	**Huoditang**	**Maoxian**	**Mt. Gongga**	**Mt. Ailao**	**Xishuangbanna**	**Jianfengling**	$F_{(8,26)}$	***p***
**pH**	5.02±0.038	6.85±0.015	8.12±0.001	5.9±0.001	5.6±0.127	6.2±0.001	5.94±0.047	5.07±0.088	6.3±0.009	293.147	<0.001
**BD (g/cm3)**	0.94±0.001	1.02±0.061	0.9±0.003	1.2±0.001	1.1±0.001	0.81±0.001	1.12±0.042	1.08±0.001	1.32±0.049	28.227	<0.001
**Sand (%)**	63.77±3.18	89.35±0.001	14.79±1.895	49.6±0.001	26.33±0.537	33.07±1.445	24.02±0.884	47.7±0.133	50.98±0.001	281.455	<0.001
**Silt (%)**	23.93±2.088	9.65±0.001	60.93±1.804	49.87±0.001	64.14±2.134	4.97±0.12	62.58±0.832	29.53±0.324	41.22±0.001	357.653	<0.001
**Clay (%)**	12.3±1.185	1±0.001	24.28±1.079	0.53±0.001	9.52±1.993	61.97±1.328	13.4±1.481	22.78±0.294	7.8±0.001	298.839	<0.001
**shannon**	7.78±0.104	8.3±0.11	7.93±0.032	7.43±0.238	7.83±0.07	8.24±0.119	8.11±0.214	6.02±0.246	7.58±0.119	19.465	<0.001
**Fungi** (log_{10} copy number/g)	5.12±0.044	4.18±0.248	4.5±0.117	4.39±0.184	4.06±0.058	3.62±0.193	4.34±0.244	4.07±0.09	3.14±0.132	11.746	<0.001
**Bacteria** (log_{10} copy number/g)	7.9±0.062	6.96±0.196	8.06±0.121	7.27±0.609	7.19±0.119	6.62±0.49	7.43±0.297	7.74±0.193	5.58±0.244	5.997	0.001
**F: B**	0.65±0.012	0.6±0.02	0.56±0.012	0.61±0.027	0.57±0.009	0.55±0.031	0.58±0.009	0.53±0.017	0.56±0.015	4.071	0.006
**SOC(g/kg)**	99.33±6.04	46.21±5.54	39.37±5.736	40.95±4.549	28.6±2.834	38.92±6.083	36.37±2.873	20.38±0.367	33.76±0.603	26.197	<0.001
**C: N**	31.34±0.556	33.7±6.034	27.45±7.289	21.53±1.999	17.67±1.169	17.06±2.608	14.23±0.01	11.83±0.864	14.66±0.787	5.514	0.001
**labile C (%)**	30.41±2.544	28.7±2.067	38.66±4.129	22.8±6.778	51.64±2.541	58.15±9.079	49.48±0.792	47.27±5.379	39.96±3.304	6.2	0.001
**Recalcitrant C (%)**	69.59±2.544	71.3±2.067	61.34±4.129	77.2±6.778	48.36±2.541	41.85±9.079	50.52±0.792	52.73±5.379	60.04±3.304	6.2	0.001
**Alky C (%)**	13.4±0.531	9.52±0.092	5.86±0.306	13.65±1.042	1.94±0.153	10.96±0.139	7.85±0.104	3.51±0.294	8.83±0.095	92.413	<0.001
**Oalkyl C (%)**	31.12±1.449	31.16±0.165	31.71±2.286	29.36±3.548	34.23±1.599	35.28±0.3	31.44±0.387	38.01±0.393	33.68±0.84	2.691	0.039
**Alky C: Oalkyl C**	0.43±0.038	0.31±0.003	0.19±0.023	0.47±0.02	0.06±0.006	0.31±0.001	0.25±0.001	0.09±0.006	0.26±0.009	68.912	<0.001
**Aromatic C (%)**	47.62±0.805	53.13±0.346	57.79±0.069	47.89±4.067	57.65±0.205	43.97±0.188	58.37±1.648	52.96±0.407	54.37±0.684	11.429	<0.001
**Carboxy C (%)**	5.95±1.671	5.36±0.009	4±1.542	7.73±0.849	2.91±0.716	8.92±0.358	1.67±0.964	3.68±0.199	2.45±0.133	7.175	<0.001
**Carbonyl C (%)**	1.91±0.052	0.84±0.095	0.64±0.37	1.37±0.326	3.28±0.93	0.86±0.268	0.67±0.193	1.84±0.479	0.67±0.193	4.677	0.003

Mt., mountain; BD, bulk density; C: N, soil organic carbon (C) to nitrogen ratio; F: B ratio, fungi: bacteria; A:O, Alky C: Oalkyl C. Data are presented as mean ± standard errors of the estimated effect sizes (n = 3, biologically independent samples). Source data are provided as a Source Data file. Some information regarding the trends of soil basic physio-chemical and biological in forests across biomes have been showed in our previous study *(**Ren, C. J. et al**. Microbial traits determine soil C emission in response to fresh carbon inputs in forests across biomes. Global Change Biology 28, 1516-1528, doi:10.1111/gcb.16004 (2022))*;

Supplementary Table 3 | Relationships between microbial physiological traits and mean annual temperature at different measuring temperature. MAT, mean annual temperature; Mass-specific growth is growth per unit microbial biomass carbon, while mass-specific respiration is respiration per unit microbial biomass carbon. Source data are provided as a Source Data file (n = 3, biologically independent samples).

**Physiological traits**	**Measuring temperature (°C)**	**Equation**	R^2	***P***
Carbon use efficiency	5	y=-0.0185x+0.7637	0.82	<0.001
	10	y=-0.0178x+0.7560	0.80	<0.001
	15	y=-0.0134x+0.68	0.79	<0.001
	20	y=-0.0144x+0.7048	0.81	<0.001
	25	y=-0.0078x+0.6323	0.49	<0.001
	30	y=-0.0091x+0.6261	0.68	<0.001
Mass-specific growth	5	y=-0.0169x+0.8216	0.52	<0.001
	10	y=-0.0398x+1.3729	0.44	<0.001
	15	y=-0.0208x+1.0935	0.14	0.052
	20	y=-0.0590x+1.9619	0.54	<0.001
	25	y=-0.0387x+2.1987	0.13	0.061
	30	y=-0.1087x+4.0359	0.39	<0.001
Mass-specific respiration	5	y=0.0250x+0.2398	0.69	<0.001
	10	$y=-0.0054x^2+0.1561x+0.1424$	0.67	<0.001
	15	y=0.0260x+0.4515	0.35	<0.001
	20	$y=-0.0051x^2+0.1418x+0.2984$	0.54	<0.001
	25	y=0.0200x+1.1793	0.11	0.091
	30	$y=-0.0101x^2+0.2538x+1.2693$	0.44	<0.001

Supplementary Table 4 | Akaike information criterions for linear and exponential functions in fitting the relationships between microbial physiological traits and measuring temperature. Source data are provided as a Source Data file.

Microbial physiology	Study sites	Linear function	Exponential function
Carbon use efficiency	Mt. Maoer	-65.2473	-60.7426
	Mt. Dongling	-58.0453	-50.7939
	Fuxian	-62.8806	-50.0061
	Huoditang	-76.3749	-63.2925
	Maoxian	-57.5239	-53.5315
	Mt. Gongga	-78.6382	-72.5459
	Mt. Ailao	-92.6791	-85.8054
	Xishuangbanna	-58.148	-47.9978
	Jianfengling	-57.0253	-53.7097
Growth	Mt. Maoer	262.2253	253.3677
	Mt. Dongling	273.6244	271.0556
	Fuxian	260.6953	255.3485
	Huoditang	223.7732	216.4848
	Maoxian	229.6291	206.5959
	Mt. Gongga	217.2389	211.4432
	Mt. Ailao	209.7603	200.3569
	Xishuangbanna	198.3124	196.5032
	Jianfengling	208.7361	203.7676
Respiration	Mt. Maoer	245.8891	218.701
	Mt. Dongling	241.0022	228.7135
	Fuxian	248.1128	237.4673
	Huoditang	218.2446	191.1666
	Maoxian	227.672	218.9297
	Mt. Gongga	221.8945	217.3716
	Mt. Ailao	218.1439	208.5814
	Xishuangbanna	199.8814	197.786
	Jianfengling	208.6679	199.0898

Supplementary Table 5 | Principal components analysis of climates, soil properties, microbial community, and microbial carbon decomposition genes. Loadings and correlations for each factor with the first component are shown. ***, $P < 0.001$; **, $P < 0.01$; *, $P < 0.05$. Source data are provided as a Source Data file.

	**Factors**	**Loadings (%)**	**Correlations**
**Climate factors**	Mean annual precipitation	0.707	0.902***
	Mean annual temperature	0.707	0.621***
	Proportion of variance	92.17%	
**Soil properties**	Sand	0.707	0.910***
	Silt	-0.707	-0.910***
	Proportion of variance	82.83%	
**Soil C quality**	Labile carbon	0.404	0.651***
	Alky carbon	-0.477	-0.866***
	Oalkyl carbon	0.420	0.741***
	Alky carbon:Oalkyl carbon	-0.535	-0.953***
	Carboxy carbon	-0.382	-0.699***
	Proportion of variance	63.40%	
**Microbial community**	Shannon	0.635	0.878***
	Fungi:bacteria	0.473	0.656***
	Proteobacteria	0.611	0.798***
	Proportion of variance	64.17%	
	Shannon	0.635	0.878***
**Microbial C decomposition genes**	Lipids	0.612	-0.900***
	Lignin	0.629	-0.933***
	Aminosugars	-0.478	0.705***
	Proportion of variance	75.81%	

Supplementary Table 6 | The *P* values of correlations between environmental factors and microbial carbon use efficiency and its thermal sensitivity. Microbial carbon use efficiency (CUE) and its thermal sensitivity (CUE_T) were measured at 5 (CUE_5), 10 (CUE_{10}), 15 (CUE_{15}), 20 (CUE_{20}), 25 (CUE_{25}), and 30 °C (CUE_{30}). Two-sided statistical tests were used to evaluate the data. Significant *P* values (< 0.05) are shown in bold.

	CUE_5	CUE_{10}	CUE_{15}	CUE_{20}	CUE_{25}	CUE_{30}	CUE_T
MAP	**0.001**	**<0.001**	**<0.001**	**<0.001**	**0.015**	**<0.001**	**0.035**
MAT	**<0.001**	**<0.001**	**<0.001**	**<0.001**	**<0.001**	**<0.001**	**<0.001**
Bulk density	0.059	0.059	**0.039**	**0.038**	0.170	**0.039**	0.254
pH	0.155	0.201	0.378	0.129	0.210	0.387	**0.019**
Sand	**0.011**	**0.005**	**0.001**	**0.004**	**<0.001**	**0.026**	0.056
Silt	0.288	0.192	0.072	0.142	**0.016**	0.265	0.640
Clay	0.097	0.089	0.115	0.111	0.186	0.229	0.082
Labile C	**<0.001**	**<0.001**	**<0.001**	**<0.001**	**0.014**	**0.033**	**<0.001**
Recalcitrant C	**<0.001**	**<0.001**	**<0.001**	**<0.001**	**0.014**	**0.033**	**<0.001**
Alky C	**0.004**	**0.010**	**0.006**	**0.005**	**0.037**	0.357	**<0.001**
Oalkyl C	**0.003**	**0.007**	**0.020**	**0.011**	0.385	**0.043**	**0.001**
Alky C: Oalkyl C	**0.001**	**0.003**	**0.003**	**0.002**	**0.024**	0.199	**<0.001**
Aromatic C	0.259	0.239	0.106	0.188	**0.043**	0.605	0.342
Carboxy C	0.058	0.032	**0.028**	0.051	0.083	0.098	0.085
Carbonyl C	0.791	0.747	0.928	0.783	0.599	0.217	0.131
Fungi	**<0.001**	**<0.001**	**<0.001**	**<0.001**	**0.005**	**<0.001**	**0.003**
Bacteria	**0.046**	**0.047**	0.068	0.052	0.312	**0.043**	0.132
Fungi: Bacteria	**<0.001**	**0.001**	**0.003**	**0.002**	**<0.001**	**0.010**	**0.006**
Pro	0.953	0.843	0.827	0.991	0.802	0.806	0.737
Aci	0.092	0.271	0.312	0.142	0.965	0.649	**0.026**
Act	0.946	0.694	0.766	0.907	0.201	0.966	0.768
Ver	**0.030**	0.098	0.071	0.066	**0.005**	0.262	0.085
Pla	0.945	0.870	0.780	0.908	0.807	0.342	0.527
Chl	0.107	0.246	0.444	0.152	0.827	0.302	0.056
Gem	0.076	0.164	0.205	0.084	0.956	0.441	**0.017**
Mon	**0.026**	0.158	0.250	0.105	0.938	0.631	**0.001**
Dis	0.125	0.082	**0.026**	**0.047**	**0.004**	**0.024**	0.671
Pol	0.191	0.214	0.432	0.383	0.983	0.884	**0.034**
Hem	0.131	0.106	0.130	0.173	0.198	0.054	0.391
Cel	0.560	0.354	0.201	0.249	0.283	0.233	0.997
Ami	**0.008**	**0.030**	**0.043**	**0.021**	0.444	0.496	**<0.001**
Lip	**0.023**	**0.032**	**0.018**	**0.016**	0.138	0.314	**0.012**
Chi	0.066	**0.021**	**0.026**	0.057	0.102	**0.036**	0.174
Lig	**0.036**	0.059	**0.040**	**0.027**	0.115	0.327	**0.022**

Supplementary Table 7 | The *P* values and standard path coefficients of structural equation modeling for microbial carbon use efficiency. CUE_{20}, microbial carbon use efficiency measured at 20°C.

Relationships	*P*	Standard path coefficients
Soil properties ~ Climate	0.708	-0.072
Soil carbon quality ~ Climate	0.138	0.250
Soil carbon quality ~ Soil properties	0.019	-0.397
Microbial community ~ Climate	0.010	-0.411
Microbial community ~ Soil carbon quality	0.027	-0.351
Microbial carbon decomposition genes ~ Climate	0.901	-0.019
Microbial carbon decomposition genes ~ Soil properties	0.191	-0.185
Microbial carbon decomposition genes ~ Microbial community	0.024	-0.367
Microbial carbon decomposition genes ~ Soil carbon quality	0.009	0.410
CUE_{20} ~ Climate	0.000	-0.741
CUE_{20} ~ Microbial community	0.036	-0.199
CUE_{20} ~ Microbial carbon decomposition genes	0.237	-0.122
CUE_{20} ~ Soil properties	0.004	0.223
CUE_{20} ~ Soil carbon quality	0.000	-0.342

Supplementary Table 8 | The *P* values and standard path coefficients of structural equation modeling for thermal sensitivity of soil microbial carbon use efficiency. CUE_T, thermal sensitivity of soil microbial carbon use efficiency.

Relationships	*P*	Standard path coefficients
Soil properties ~ Climate	0.708	-0.072
Soil carbon quality ~ Climate	0.138	0.250
Soil carbon quality ~ Soil properties	0.019	-0.397
Microbial community ~ Climate	0.010	-0.411
Microbial community ~ Soil carbon quality	0.027	-0.351
Microbial carbon decomposition genes ~ Climate	0.901	-0.019
Microbial carbon decomposition genes ~ Soil properties	0.191	-0.185
Microbial carbon decomposition genes ~ Microbial community	0.024	-0.367
Microbial carbon decomposition genes ~ Soil carbon quality	0.009	0.410
CUE_T~ Climate	0.000	0.504
CUE_T ~ Microbial community	0.025	0.288
CUE_T~ Microbial carbon decomposition genes	0.019	0.326
CUE_T~ Soil properties	0.250	0.122
CUE_T ~ Soil carbon quality	0.000	0.555

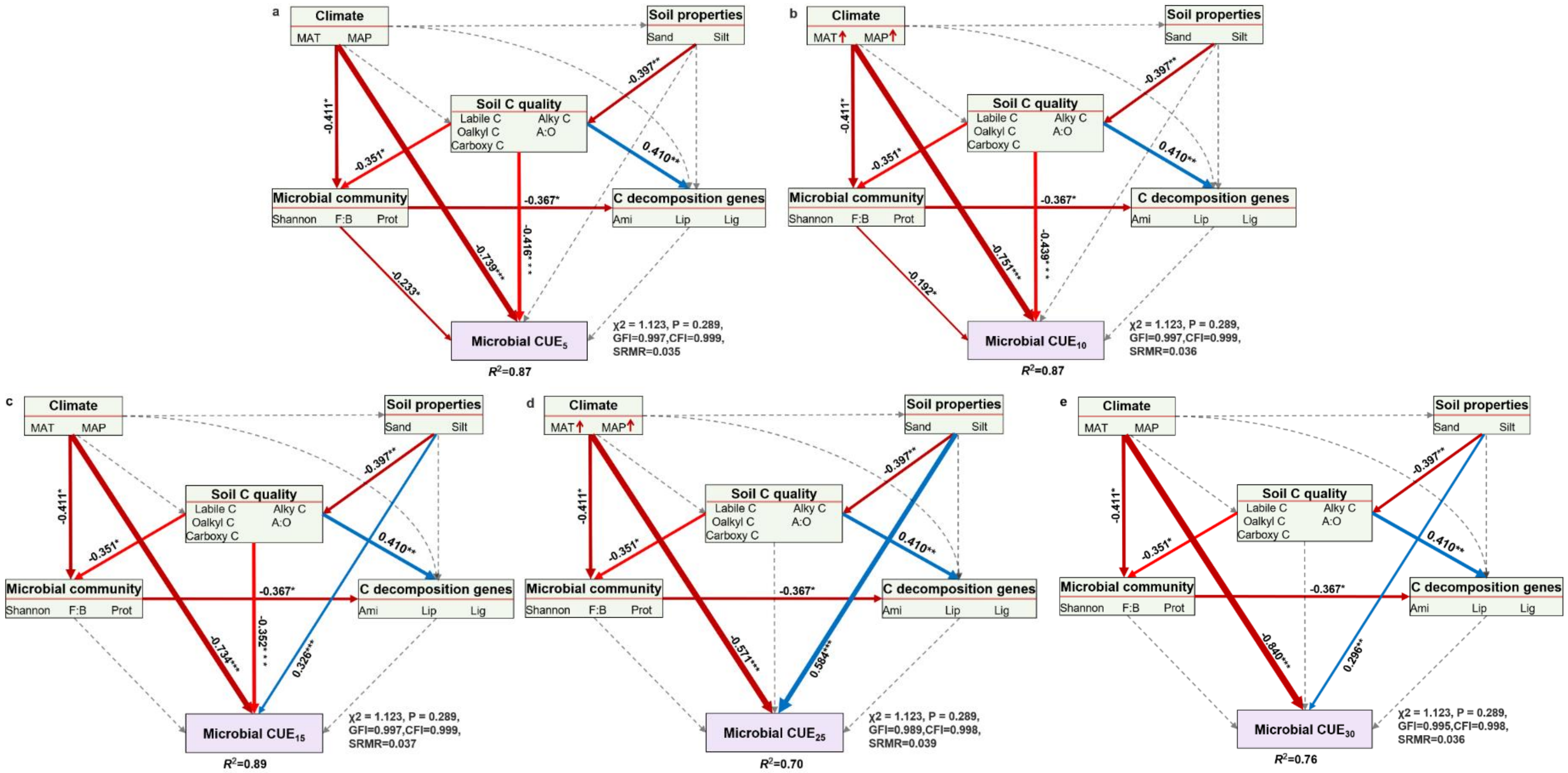

Supplementary Fig. 1 | Drivers of microbial carbon use efficiencies at 5°C, 10 °C, 15 °C, 25 °C, and 30 °C. Directed graph of the structural equation modeling showing the effects of the soil properties, soil carbon (C) quality, microbial community, and microbial C decomposition genes induced by climate on CUE_5 (a), CUE_{10} (b), CUE_{15} (c), CUE_{25} (d), and CUE_{30} (e), respectively. Single-headed arrows indicate the hypothesized direction of causation, while indicated values are the path coefficients. Blue and red arrows indicate positive and negative relationships, respectively, while grey dashed line indicate no relationships. Numbers adjacent to arrows denote standardized path coefficients, and the arrow width is proportional to the strength of the relationship. Climate, soil properties, soil C quality, micronbial community, and microbial C decomposition genes are the first components from the PCA conducted with the factors listed in the rectangles. MAT, mean annual temperature; MAP, mean annual precipitation, F:B ratio, fungi:bacteria ratio; Pro, *Proteobacteria*; Ami, *Aminosugars*; Lip, Lipids; Lig, Lignin; *, $P < 0.05$; **, $P < 0.01$; ***, $P < 0.001$. GFI, goodness fit index; CFI, comparative fit index; SRMR, standardized Root Mean Square Residual. Source data are provided as a Source Data file.

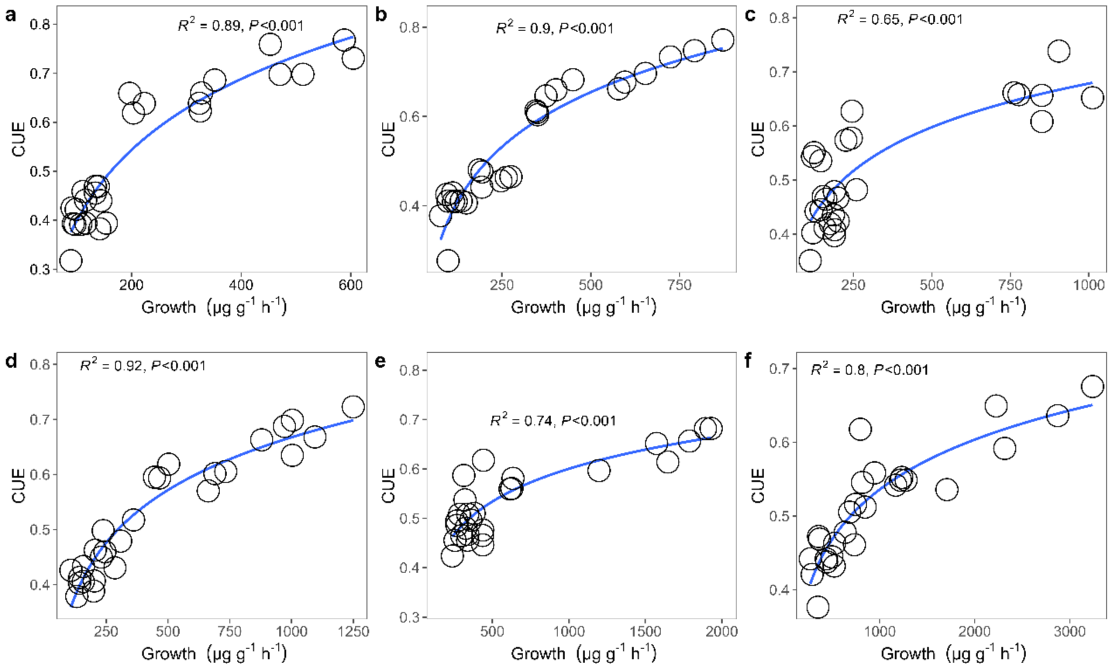

Supplementary Fig. 2 | Relationship between microbial carbon use efficiency and growth at 5°C (a), 10°C (b), 15°C (c), 20°C (d), 25°C (e), and 30°C (f). CUE, microbial carbon use efficiency. Source data are provided as a Source Data file.

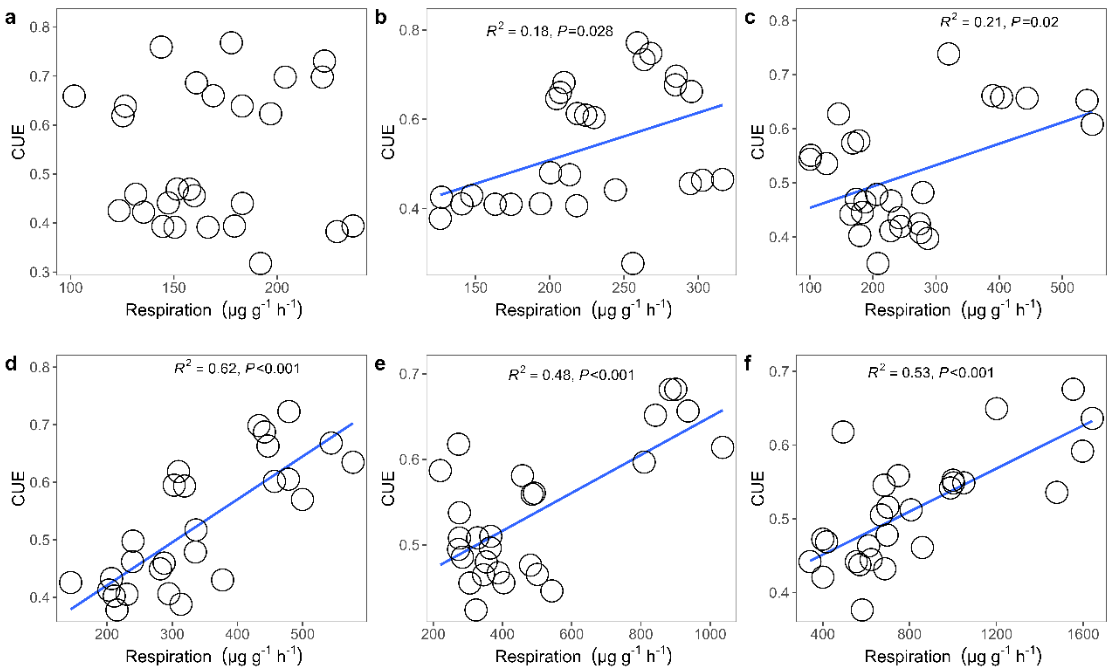

Supplementary Fig. 3 | Relationship between microbial carbon use efficiency and respiration at 5°C (a), 10°C (b), 15°C (c), 20°C (d), 25°C (e), and 30°C (f). CUE, microbial carbon use efficiency. Source data are provided as a Source Data file.

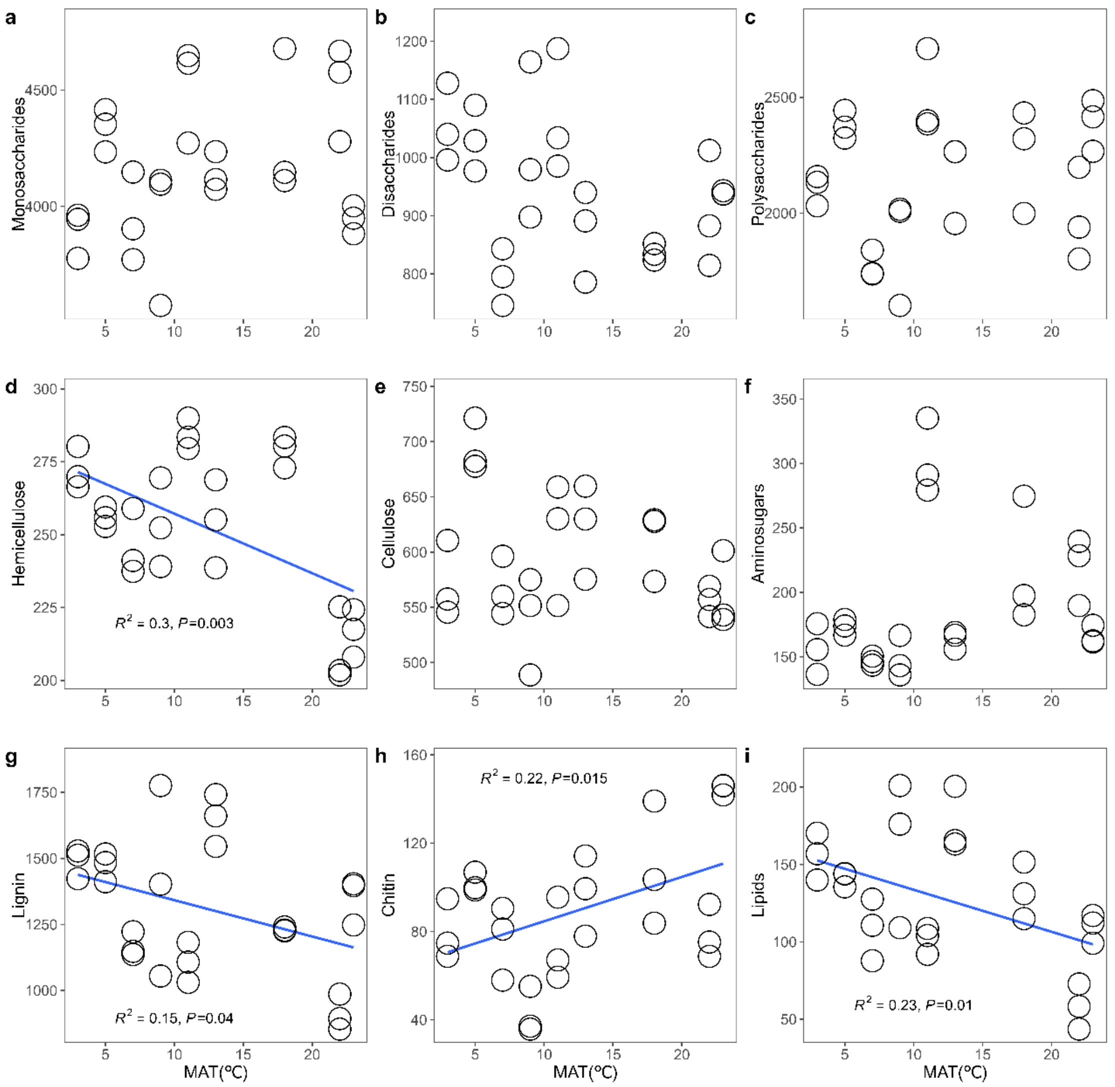

Supplementary Fig. 4 | Relationships between microbial carbon-decomposition genes and MAT. **a** Relationship between carbon-decomposition genes in degrading monosaccharides and MAT. **b** Relationship between carbon-decomposition genes in degrading disaccharides and MAT. **c** Relationship between carbon-decomposition genes in degrading polysaccharides and MAT. **d** Relationship between carbon-decomposition genes in degrading hemicellulose and MAT. **e** Relationship between carbon-decomposition genes in degrading cellulose and MAT. **f** Relationship between carbon-decomposition genes in degrading aminosugar and MAT. **g** Relationship between carbon-decomposition genes in degrading lignin and MAT. **h** Relationship between carbon-decomposition genes in degrading chitin and MAT. **i** Relationship between carbon-decomposition genes in degrading lipids and MAT. MAT, mean annual temperature. Source data are provided as a Source Data file.

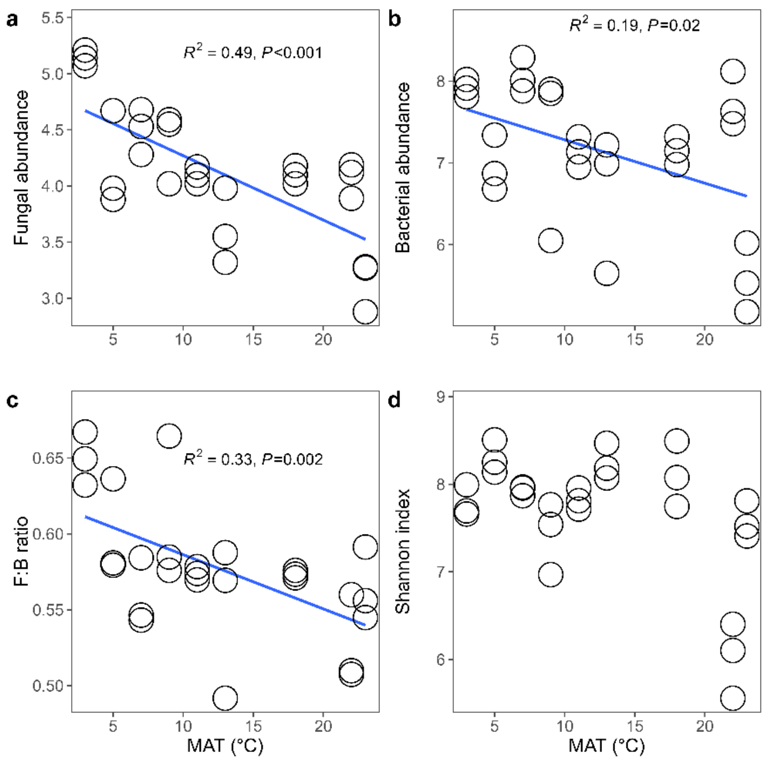

Supplementary Fig. 5 | Relationships between microbial community diversity, abundance and MAT. **a** Relationship between fungal abundance and MAT. **b** Relationship between bacterial abundance and MAT. **c** Relationship between F:B ratio and MAT. **d** Relationship between Shnnon index and MAT. MAT, mean annual temperature; F:B ratio, fungi: bacteria. Source data are provided as a Source Data file.

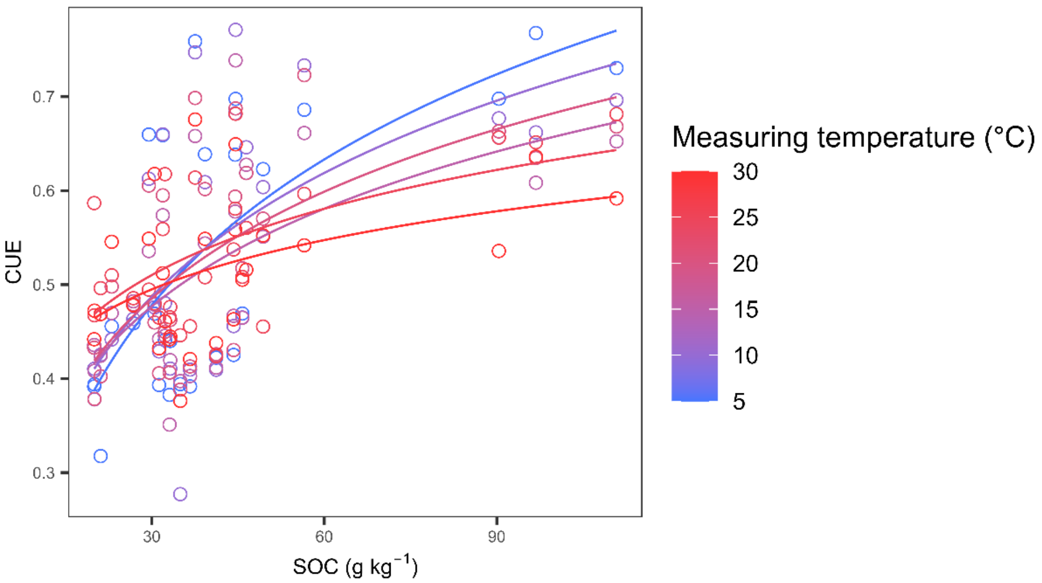

Supplementary Fig. 6 | Relationship between microbial carbon use efficiency and soil organic carbon at different measuring temperatures. SOC, soil organic carbon; CUE, microbial carbon use efficiency. Source data are provided as a Source Data file.

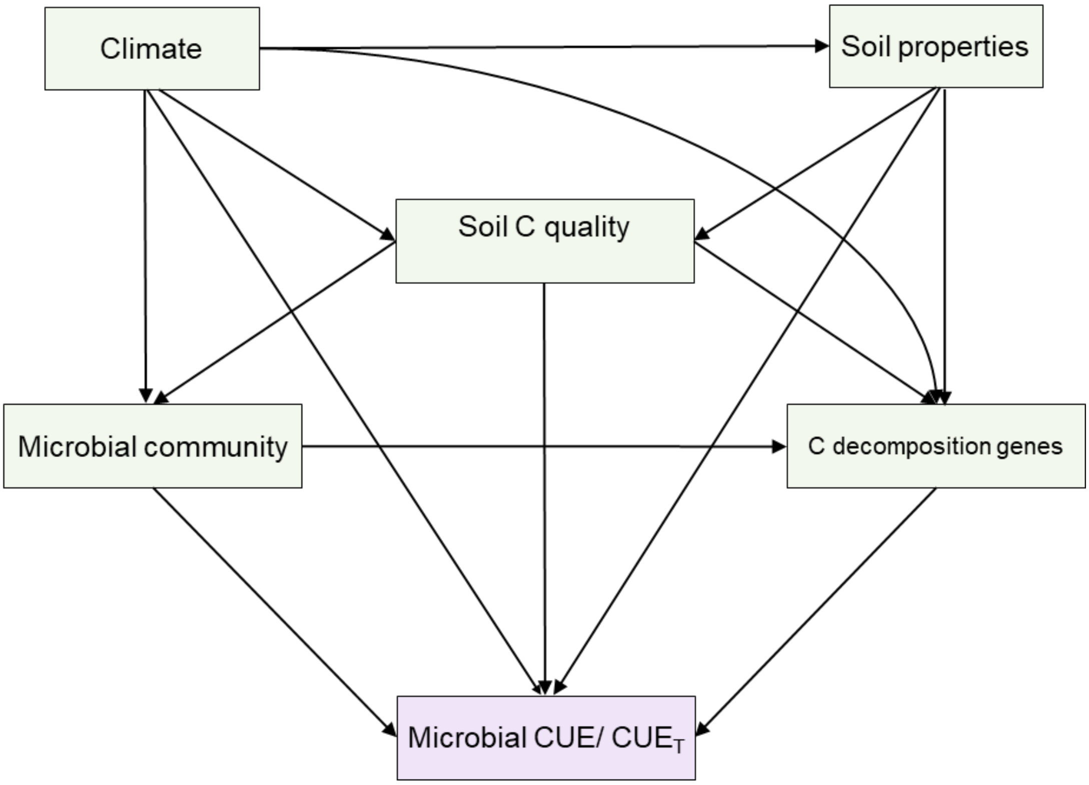

Supplementary Fig. 7 | A priori model showing the effects of climate, soil properties, soil carbon quality, microbial community structure, and microbial carbon decomposition genes on microbial carbon use efficiency and its thermal sensitivity. In the priori model, climate, soil properties, soil carbon (C) quality, microbial community structure, and microbial C decomposition genes are the first components from the principal component analysis conducted with the corresponding factors which have significant effects on microbial carbon use efficiency (CUE) and its thermal sensitivity (CUE_T).
