# Supplementary material for: Thermal sensitivity of soil microbial carbon use efficiency across forest biomes
Source: Nat Commun. 2024 Jul 25;15:6269. doi: 10.1038/s41467-024-50593-6 (PMC11272934; doi:10.1038/s41467-024-50593-6)
Supplement: Supplementary file 3 — Reporting Summary [file 41467_2024_50593_MOESM3_ESM.pdf]

nature portfolio

Corresponding author(s): Zhenghu, Gaihe, Gehong

Last updated by author(s): May 5, 2024

Reporting Summary

Nature Portfolio wishes to improve the reproducibility of the work that we publish. This form provides structure for consistency and transparency in reporting. For further information on Nature Portfolio policies, see our Editorial Policies and the Editorial Policy Checklist.

Statistics

For all statistical analyses, confirm that the following items are present in the figure legend, table legend, main text, or Methods section.

n/a	Confirmed	
☐	☒	The exact sample size (*n*) for each experimental group/condition, given as a discrete number and unit of measurement
☐	☒	A statement on whether measurements were taken from distinct samples or whether the same sample was measured repeatedly
☐	☒	The statistical test(s) used AND whether they are one- or two-sided<br>*Only common tests should be described solely by name; describe more complex techniques in the Methods section.*
☒	☐	A description of all covariates tested
☒	☐	A description of any assumptions or corrections, such as tests of normality and adjustment for multiple comparisons
☐	☒	A full description of the statistical parameters including central tendency (e.g. means) or other basic estimates (e.g. regression coefficient) AND variation (e.g. standard deviation) or associated estimates of uncertainty (e.g. confidence intervals)
☐	☒	For null hypothesis testing, the test statistic (e.g. *F*, *t*, *r*) with confidence intervals, effect sizes, degrees of freedom and *P* value noted<br>*Give P values as exact values whenever suitable.*
☒	☐	For Bayesian analysis, information on the choice of priors and Markov chain Monte Carlo settings
☒	☐	For hierarchical and complex designs, identification of the appropriate level for tests and full reporting of outcomes
☐	☒	Estimates of effect sizes (e.g. Cohen's *d*, Pearson's *r*), indicating how they were calculated

Our web collection on statistics for biologists contains articles on many of the points above.

Software and code

Policy information about availability of computer code

Data collection	The data used in this study are available in the figshare database
Data analysis	The code used in this study are available in the figshare database

For manuscripts utilizing custom algorithms or software that are central to the research but not yet described in published literature, software must be made available to editors and reviewers. We strongly encourage code deposition in a community repository (e.g. GitHub). See the Nature Portfolio guidelines for submitting code & software for further information.

Data

Policy information about availability of data

All manuscripts must include a data availability statement. This statement should provide the following information, where applicable:

- Accession codes, unique identifiers, or web links for publicly available datasets
- A description of any restrictions on data availability
- For clinical datasets or third party data, please ensure that the statement adheres to our policy

The data used in this study are available in the figshare database

Research involving human participants, their data, or biological material

Policy information about studies with human participants or human data. See also policy information about sex, gender (identity/presentation), and sexual orientation and race, ethnicity and racism.

Reporting on sex and gender	Our study was not involved in human participants, their data, or biological material
Reporting on race, ethnicity, or other socially relevant groupings	Our study was not involved in human participants, their data, or biological material
Population characteristics	Our study was not involved in human participants, their data, or biological material
Recruitment	Our study was not involved in human participants, their data, or biological material
Ethics oversight	Our study was not involved in human participants, their data, or biological material

Note that full information on the approval of the study protocol must also be provided in the manuscript.

Field-specific reporting

Please select the one below that is the best fit for your research. If you are not sure, read the appropriate sections before making your selection.

☐ Life sciences ☐ Behavioural & social sciences ☒ Ecological, evolutionary & environmental sciences

For a reference copy of the document with all sections, see nature.com/documents/nr-reporting-summary-flat.pdf

Life sciences study design

All studies must disclose on these points even when the disclosure is negative.

Sample size	
Data exclusions	
Replication	
Randomization	
Blinding	

Behavioural & social sciences study design

All studies must disclose on these points even when the disclosure is negative.

Study description	
Research sample	
Sampling strategy	
Data collection	
Timing	
Data exclusions	
Non-participation	
Randomization	

Ecological, evolutionary & environmental sciences study design

All studies must disclose on these points even when the disclosure is negative.

Study description	Here, we used the 18O-H2O tracer method at six measuring temperatures (5, 10, 15, 20, 25, and 30°C) to assess soil microbial CUE and CUET across a 3425-km north-south forest transect in China (spanning approximately 27 latitudinal degrees; see Supplementary Fig. 1 and Table 1). As potential drivers of microbial CUE and CUET, including the direct and indirect effects of climate, soil properties, C quality, microbial community structure, and the functional genes involved in C decomposition were evaluated using structural equation modeling (SEM). Climate factors studied included mean annual temperature (MAT) and mean annual precipitation (MAP), with ranges from 3.1 to 23.15 °C and 486 to 2266 mm, respectively (Supplementary Table 1). Soil properties examined included soil pH, bulk density, and texture (Supplementary Table 2). Solid-state 13C cross polarization-magic angle spinning (CP/MAS) nuclear magnetic resonance (NMR) spectroscopy and acid hydrolysis methods were utilized to quantify soil C quality. High C quality (desirable for microbes) is indicative of low molecular weight and structural complexity, but high solubility and lability. The microbial community structure was represented by microbial diversity (Shannon index), fungal abundance, bacterial abundance, the ratio of fungi to bacteria, and the relative abundance of microbial phyla. Metagenomic sequencing was employed to explore the abundance of functional genes associated with the decomposition of various C forms. The objectives of our study were to identify how microbial CUE and its thermal sensitivity change across forest biomes from tropical to temperate regions, ascertain the potential drivers of these changes, and evaluate the implications for the future development of soil C models.
Research sample	Soil samples were collected between July and August 2019. At each site, three sampling plots (50 × 50 m) were established randomly in well-protected national nature reserves to minimize the effect of anthropogenic disturbance. These sites were in areas with relatively homogeneous vegetation, strongly representative of each forest type.
Sampling strategy	At each site, three sampling plots (50 × 50 m) were established randomly in well-protected national nature reserves to minimize the effect of anthropogenic disturbance.
Data collection	Given that topsoil microbes are highly sensitive to a range of factors associated with climate change, soils from the top 10 cm were collected to study the microbial CUET from nine random locations within each plot, after surface litter removal. The nine soil samples were then combined into a composite sample to reduce soil heterogeneity in each plot.
Timing and spatial scale	Chengjie Ren took soil samples along a 3425-km north–south forest transects in China (across ~27 latitudinal degrees)
Data exclusions	No data were excluded
Reproducibility	All attempts to repeat the experiment were successful
Randomization	The study was conducted in nine forests along a 3425-km north–south transect in China. These forests included three temperate, four subtropical, and two tropical.
Blinding	Our data analysis was not involved in Blinding strategies

Did the study involve field work? ☒ Yes ☐ No

Field work, collection and transport

Field conditions	The study was conducted in nine forests along a 3425-km north–south transect in China. These forests included three temperate, four subtropical, and two tropical. The wide-ranging sampling transect led to substantial variations in soil and microbial properties; this provided an ideal natural laboratory for examining soil C-cycle processes and the mechanisms that underlie them. Along this transect, the mean annual temperature (MAT) at these sites ranged from 3.1 to 23.15°C, whereas mean annual precipitation (MAP) ranged from 486 to 2266 mm.

Location

Site	Elevation (m)	Latitude (°)	Longtitude (°)
Maoer M.	600	45.41	127.71
Dongling M.	1300	39.97	115.43
Fuxian	1035	36.11	109.63
Huoditang	1526	33.43	108.45
Maoxian	1625	31.79	104.71
Gongga M.	1726	29.65	102.11
Ailao M.	2300	23.87	103.51
Xishuangbanna	560	21.83	101.20
Jianfengling	820	18.71	108.91

Access & import/export	We have made to access habitats and to collect soil samples in a responsible manner and in compliance with local, national and international laws
Disturbance	This is no disturbance in our study

Reporting for specific materials, systems and methods

We require information from authors about some types of materials, experimental systems and methods used in many studies. Here, indicate whether each material, system or method listed is relevant to your study. If you are not sure if a list item applies to your research, read the appropriate section before selecting a response.

Materials & experimental systems

n/a	Involved in the study
☐	☐ Antibodies
☐	☐ Eukaryotic cell lines
☐	☐ Palaeontology and archaeology
☐	☒ Animals and other organisms
☐	☐ Clinical data
☐	☐ Dual use research of concern
☐	☐ Plants

Methods

n/a	Involved in the study
☐	☒ ChIP-seq
☐	☐ Flow cytometry
☐	☐ MRI-based neuroimaging

Antibodies

Antibodies used	This study did not involved in antibodies
Validation	This study did not involved in antibodies

Eukaryotic cell lines

Policy information about cell lines and Sex and Gender in Research

Cell line source(s)	This study did not involved in cell line
Authentication	This study did not involved in cell line
Mycoplasma contamination	This study did not involved in cell line
Commonly misidentified lines (See ICLAC register)	This study did not involved in cell line

Palaeontology and Archaeology

Specimen provenance	This study did not involved in palaeontology and archaeology
Specimen deposition	This study did not involved in palaeontology and archaeology
Dating methods	This study did not involved in palaeontology and archaeology

☒ Tick this box to confirm that the raw and calibrated dates are available in the paper or in Supplementary Information.

Ethics oversight	This study did not involved in Ethics oversight

Note that full information on the approval of the study protocol must also be provided in the manuscript.

Animals and other research organisms

Policy information about studies involving animals; ARRIVE guidelines recommended for reporting animal research, and Sex and Gender in Research

Laboratory animals	This study did not involved in laboratory animals
Wild animals	This study did not involved in Wild animals
Reporting on sex	This study did not involved in reporting on sex
Field-collected samples	The broad sampling transect results in great variations of soil and microbial properties, providing an ideal natural laboratory to explore the soil C cycling processes and their underlying mechanism. Along this transect, the mean annual temperature (MAT) at

	these sites ranged from 3.1 to 23.15°C, whereas mean annual precipitation (MAP) ranged from 486 to 2266 mm.
Ethics oversight	This study did not involved in Ethics oversight

Note that full information on the approval of the study protocol must also be provided in the manuscript.

Clinical data

Policy information about clinical studies

All manuscripts should comply with the ICMJE guidelines for publication of clinical research and a completed CONSORT checklist must be included with all submissions.

Clinical trial registration	This study did not involved in clinical trial
Study protocol	This study did not involved in clinical trial
Data collection	This study did not involved in clinical trial
Outcomes	This study did not involved in clinical trial

Dual use research of concern

Policy information about dual use research of concern

Hazards

Could the accidental, deliberate or reckless misuse of agents or technologies generated in the work, or the application of information presented in the manuscript, pose a threat to:

No	Yes	
☒	☐	Public health
☒	☐	National security
☒	☐	Crops and/or livestock
☒	☐	Ecosystems
☒	☐	Any other significant area

Experiments of concern

Does the work involve any of these experiments of concern:

No	Yes	
☒	☐	Demonstrate how to render a vaccine ineffective
☒	☐	Confer resistance to therapeutically useful antibiotics or antiviral agents
☒	☐	Enhance the virulence of a pathogen or render a nonpathogen virulent
☒	☐	Increase transmissibility of a pathogen
☒	☐	Alter the host range of a pathogen
☒	☐	Enable evasion of diagnostic/detection modalities
☒	☐	Enable the weaponization of a biological agent or toxin
☒	☐	Any other potentially harmful combination of experiments and agents

Plants

Seed stocks	This study did not involved in plants
Novel plant genotypes	This study did not involved in plants
Authentication	This study did not involved in plants

ChIP-seq

Data deposition

☒ Confirm that both raw and final processed data have been deposited in a public database such as GEO.

☐ Confirm that you have deposited or provided access to graph files (e.g. BED files) for the called peaks.

Data access links<br>*May remain private before publication.*	All sequences associated with this study are available from the Sequence Read Archive under accession numbers PRJNA977727
Files in database submission	All sequences associated with this study are available from the Sequence Read Archive under accession numbers PRJNA977727
Genome browser session<br>(e.g. UCSC)	All sequences associated with this study are available from the Sequence Read Archive under accession numbers PRJNA977727

Methodology

Replicates	To obtain sufficient DNA for the shotgun metagenomic sequencing and to guarantee the representation of forest soil, six replicates were conducted from each soil sample.
Sequencing depth	10G each soil samples
Antibodies	This study did not involved in antibodies
Peak calling parameters	This study did not involved in peak calling parameters
Data quality	To improve the reliability and quality of subsequent analysis, the sequencing reads were filtered as previously described, which included removing the adapter sequences, trimming the reads, and discarding the quality-trimmer reads that were below than 50 bp or containing N (ambiguous bases). For the utilization of high-quality reads, the megahit software was used to assemble the mixed sequence of all samples, and thus the larger database of contigs and Scaffolds were generated. It is noted that not all sample data are mixed and sequenced, instead, each sample is individually assembled first, then mixing the unused reads from the single spell together to obtain more contigs data.
Software	The MetaGeneMark was used to predict the genes in the contigs (longer than 200bp), and the per-base coverage depth across all contigs was calculated by mapping raw reads from each sample

Flow Cytometry

Plots

Confirm that:

☐ The axis labels state the marker and fluorochrome used (e.g. CD4-FITC).

☐ The axis scales are clearly visible. Include numbers along axes only for bottom left plot of group (a 'group' is an analysis of identical markers).

☐ All plots are contour plots with outliers or pseudocolor plots.

☐ A numerical value for number of cells or percentage (with statistics) is provided.

Methodology

Sample preparation	
Instrument	
Software	
Cell population abundance	
Gating strategy	

☐ Tick this box to confirm that a figure exemplifying the gating strategy is provided in the Supplementary Information.

Magnetic resonance imaging

Experimental design

Design type

Design specifications

Behavioral performance measures

Acquisition

Imaging type(s)

Field strength

Sequence & imaging parameters

Area of acquisition

Diffusion MRI ☐ Used ☒ Not used

Preprocessing

Preprocessing software

Normalization

Normalization template

Noise and artifact removal

Volume censoring

Statistical modeling & inference

Model type and settings

Effect(s) tested

Specify type of analysis: ☐ Whole brain ☐ ROI-based ☐ Both

Statistic type for inference

(See Eklund et al. 2016)

Correction

Models & analysis

n/a	Involved in the study
☐	☐ Functional and/or effective connectivity
☐	☐ Graph analysis
☐	☐ Multivariate modeling or predictive analysis

Functional and/or effective connectivity: Pearson analysis

Graph analysis: This study did not involved in graph analysis

Multivariate modeling and predictive analysis
